# Human-derived bacterial strains mitigate colitis via modulating gut microbiota and repairing intestinal barrier function in mice

**DOI:** 10.1186/s12866-024-03216-5

**Published:** 2024-03-23

**Authors:** Juanjuan Dai, Mingjie Jiang, Xiaoxin Wang, Tao Lang, Leilei Wan, Jingjing Wang

**Affiliations:** 1 Shanghai Key Laboratory of Pancreatic Diseases, Institute of Translational Medicine, Shanghai General Hospital, Shanghai Jiao Tong University School of Medicine, Shanghai, China; 2 Department of Intensive Care Unit, State Key Laboratory of Oncology in South China, Collaborative Innovation Center for Cancer Medicine, Sun Yat-sen University Cancer Center, Guangzhou, 510060 P.R. China; 3 Department of Head and Neck Surgery, State Key Laboratory of Oncology in South China, Collaborative Innovation Center for Cancer Medicine, Sun Yat-sen University Cancer Center, Guangzhou, 510060 P.R. China; 4 Department of Stomatology, Shanghai General Hospital, Shanghai Jiao Tong University School of Medicine, Shanghai, China

**Keywords:** Gut microbiota, Gut barrier dysfunction, Colitis, Human-derived strain, Short chain fatty acids

## Abstract

**Background:**

Unbalanced gut microbiota is considered as a pivotal etiological factor in colitis. Nevertheless, the precise influence of the endogenous gut microbiota composition on the therapeutic efficacy of probiotics in colitis remains largely unexplored.

**Results:**

In this study, we isolated bacteria from fecal samples of a healthy donor and a patient with ulcerative colitis in remission. Subsequently, we identified three bacterial strains that exhibited a notable ability to ameliorate dextran sulfate sodium (DSS)-induced colitis, as evidenced by increased colon length, reduced disease activity index, and improved histological score. Further analysis revealed that each of *Pediococcus acidilactici* CGMCC NO.17,943, *Enterococcus faecium* CGMCC NO.17,944 and *Escherichia coli* CGMCC NO.17,945 significantly attenuated inflammatory responses and restored gut barrier dysfunction in mice. Mechanistically, bacterial 16S rRNA gene sequencing indicated that these three strains partially restored the overall structure of the gut microbiota disrupted by DSS. Specially, they promoted the growth of *Faecalibaculum* and *Lactobacillus murinus*, which were positively correlated with gut barrier function, while suppressing *Odoribacter*, *Rikenella*, *Oscillibacter* and *Parasutterella*, which were related to inflammation. Additionally, these strains modulated the composition of short chain fatty acids (SCFAs) in the cecal content, leading to an increase in acetate and a decrease in butyrate. Furthermore, the expression of metabolites related receptors, such as receptor G Protein-coupled receptor (GPR) 43, were also affected. Notably, the depletion of endogenous gut microbiota using broad-spectrum antibiotics completely abrogated these protective effects.

**Conclusions:**

Our findings suggest that selected human-derived bacterial strains alleviate experimental colitis and intestinal barrier dysfunction through mediating resident gut microbiota and their metabolites in mice. This study provides valuable insights into the potential therapeutic application of probiotics in the treatment of colitis.

**Supplementary Information:**

The online version contains supplementary material available at 10.1186/s12866-024-03216-5.

## Background

Ulcerative colitis (UC), an inflammatory disease, arises from a confluence of etiological factors, including genetic predisposition, compromised gut barrier integrity, gut microbiota imbalance, and immune system dysregulation [1]. However, the exact pathogenesis is not well understood in detail. Lately, accumulating evidences suggest that dysbiosis of gut microbiota plays a key role in the onset and progression of colitis [1]. First of all, in dextran sulfate sodium (DSS)-induced mouse colitis model, conventional mice develop colitis, while germ-free mice exhibit minimal or no gut inflammation, highlighting the potential role of gut microbiota as a whole in gut inflammation [2]. Furthermore, when germ-free mice are colonized with microbiota from UC patients, they manifest more severe colitis compared to those colonized with microbiota from healthy individuals, underscoring the critical role of gut microbiota in colitis pathogenesis [3]. Secondly, in detail, the gut microbiota of UC patients is characterized by reduced bacterial diversity [4] and an altered composition, with an increase in opportunistic pathogens such as *Enterobacteriaceae* and a decrease in potential beneficial bacteria, including *Faecalibacterium Prausnitzii* and *Roseburia hominis* in humans and/or mice [5–7]. This dysbiosis can exacerbate colitis by promoting both innate and adaptive immune responses. For instance, it can lead to the accumulation of the innate immune adaptor STING in myeloid cells in mice, and stimulate dendritic cells and T cells through IL-6 in patients, further perpetuating the inflammatory process [8–10]. These findings indicate that gut microbiota represents a promising therapeutic target for UC.

Indeed, various strategies directly targeting gut microbiota, such as probiotics, fecal microbiota transplantation (FMT) and bacterial consortia have shown promise in ameliorating colitis in clinical trials and pre-clinical animal models [11–13]. The effective strains used in these interventions, either alone or in combination, primarily belonged to *Bifidobacterium* spp., *Lactobacillus* spp., as well as the gut commensals such as *Akkermansia muciniphila*, *Faecalibacterium prausnitzii*, *Streptococcus faecalis*, *Clostridium butyricum*, and *Bacillus mesentericus* [12, 14–18]. These bacterial strains exert their beneficial effects through multiple mechanisms. Firstly, they can directly attenuate gut inflammation by suppressing proinflammatory responses or promoting anti-inflammatory responses [8, 12, 19]. Secondly, they can enhance intestinal barrier function to prevent the initiation of colitis. For example, *L. plantarum* has been shown to reduce gut barrier disruption by upregulating the expression of tight junction proteins ZO-1 and Occludin [20]. Thirdly, these strains can modulate the composition and metabolic activity of gut microbiota, leading to further mitigation of inflammation. For example, the bacterial-host co-metabolite isoallolithocholic acid acts as a signaling molecule to promote the differentiation of Treg cells and reduce inflammation, while the bacterial metabolite butyrate augments the size and function of Treg cells, conferring protection against colitis [21–23]. However, despite the observed amelioration of colitis symptoms and concomitant alterations in endogenous gut microbiota following probiotic administration, it is still unclear whether the protective effects of these bacterial strains are directly attributed to the probiotics themselves or mediated through changes in the endogenous gut microbiota.

Although several effective strains have been identified in mouse models, there are currently limited recommended bacterial strains for the treatment of UC patients, with *Escherichia coli* Nissle being a notable exception [24]. Although the results of FMT in UC patients appear promising [13], the complexity of donor feces and the lack of standardized healthy donors have significantly hindered its widespread application. Consequently, there has been a growing focus on the role of specific bacterial strains. This has been facilitated by the analysis of fecal and colonic mucosal samples from FMT responders and non-responders, as well as their corresponding healthy donors [25, 26]. For example, *Bacteroides* in donor stools and the enrichment of *Eubacterium hallii* and *Roseburia inulivorans* in UC patients have been associated with remission [25]. Conversely, *Streptococcus* [25] and *Ruminococcus gnavus* [26] in donor stools have been linked to a poor response in UC patients. These observations further address the importance of developing more specific and standard microbe-based therapy for UC.

To identify novel potential effective bacterial strains, we isolated bacterial strains from the feces of a healthy donor and a patient with colitis in remission. Through mouse models induced by DSS and trinitrobenzoic sulphonic acid (TNBS), we successfully identified three strains that exhibited notable influence on gut inflammation and barrier function. Subsequently, we employed high-throughput sequencing, bioinformatics and statistical analysis to identify specific bacterial taxa changes after administration of these three strains. Remarkably, we observed significant associations between these changes and gut barrier function, as well as inflammation. Furthermore, we performed gas chromatography (GC)-mass spectrometry (MS) to pinpoint bacterial metabolites that contributed to the alleviation of colitis symptoms. Additionally, we conducted experiments using antibiotics to ablate gut microbiota, aiming to elucidate the necessity of endogenous microbiota in mediating the therapeutic effects of these strains on colitis. These results provided direct evidences for the pivotal role of endogenous gut microbiota on the protective effects for colitis mediated by human-derived bacteria. Notably, the identified endogenous functional bacterial strains could be used as novel candidates for the prevention and treatment of colitis. Our results provide novel insight of the role of endogenous gut microbiota modulation in bacteria-dependent amelioration of colitis.

## Results

### Human-derived bacterial strains effectively alleviated experimental colitis in mice

To explore whether and which human gut bacterial strains can potentially be used to ameliorate the symptoms of ulcerative colitis, we firstly isolated bacterial strains from the fecal samples of donor 1- a healthy infant and donor 2- a patient with colitis in remission. The gut microbiota composition of both donors were shown in Additional file 1. More than 400 strains were successfully anaerobically cultured. Through 16S rRNA gene PCR and sequencing, we identified 21 bacterial species in total. Based on the species abundance, we randomly selected 1–5 representative strains of each specie for further functional analysis, narrowing our focus to 29 bacterial strains. To assess their anti-inflammatory potential, these strains were co-cultured with human colonic epithelial cell HT-29 and inflammatory cytokines in the supernatant including IL-1β, IL-6, IL-10 were detected using ELISA. Decreased IL-1β and IL-6 level, and increased IL-10 level were considered as anti-inflammatory markers. This led to the selection of five promising anti-inflammatory human gut derived strains (Additional file 2). 16S rRNA gene sequencing and mass spectrum analysis revealed them to be *Pediococcus acidilactici* (PA; CGMCC NO. 17,943), *Enterococcus faecium* (EF; CGMCC NO. 17,944), *Escherichia coli* (EC; CGMCC NO. 17,945), *Bifidobacterium bifidus* (BB), and *Escherichia coli* 2 (EC2) (Additional file 1). Among them, PA and EF were from donor 1 (the healthy infant), and BB, EC, and EC2 were from donor 2 (the colitis patient).

**Fig. 1:**
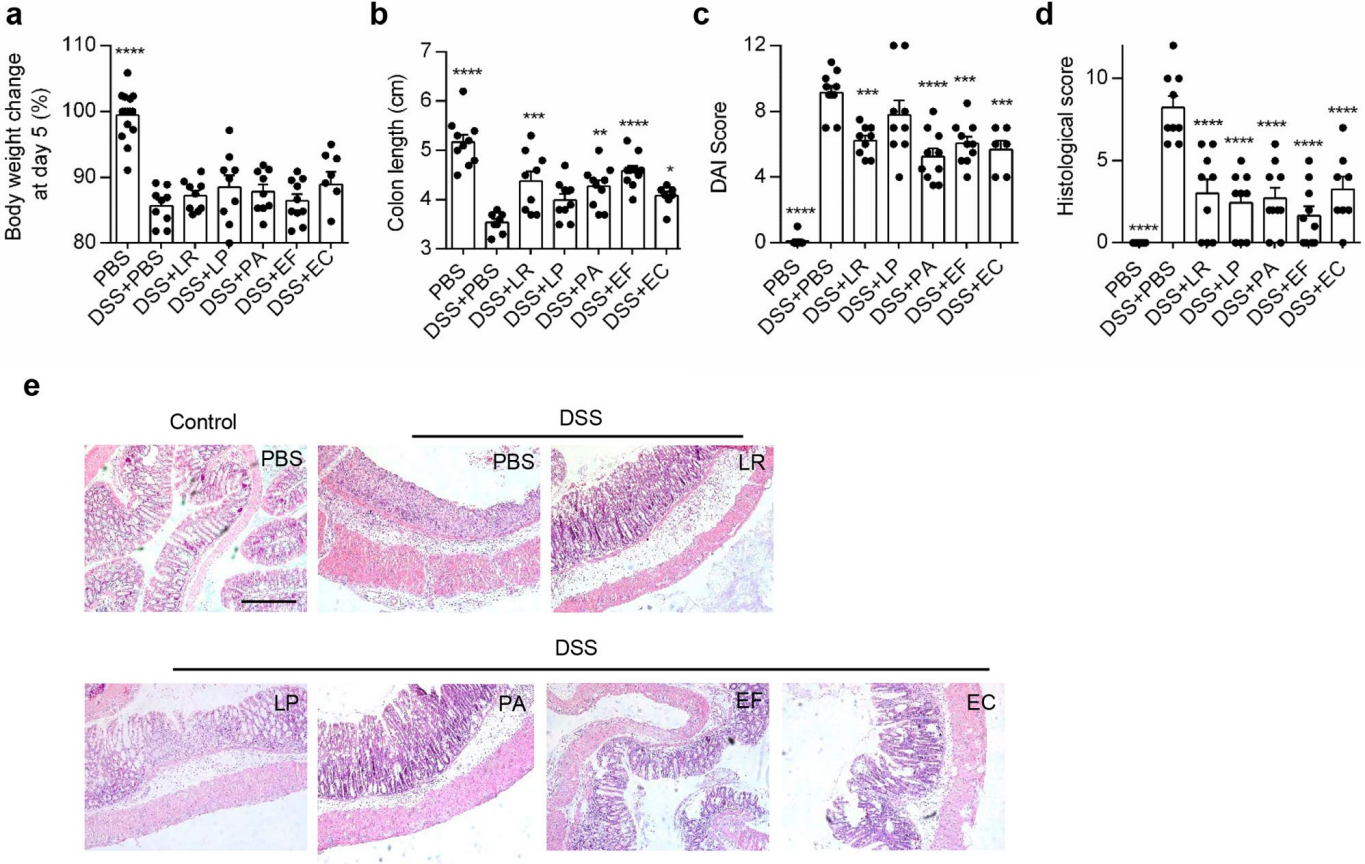
Human-derived bacterial strains effectively alleviated the severity of DSS-induced colitis in vivo. (**a**) Percentage of body weight changes at day 5 after DSS administration. (**b**) Colon length of mice at day 5 after DSS administration. (**c**) Quantification of the DAI score of mice at day 5 after DSS administration. (**d**) Quantification of histological scores of colon tissue. (**e**) Representative images showing H&E staining of colonic tissue in DSS-induced colitis mice. Scale bar = 100 μm. *n* = 5–10 mice pr group. *P < 0.05, **P < 0.01, ***P < 0.001, ****P < 0.0001 vs. DSS + PBS group by ANOVA followed by LSD post hoc test. LR: *Lactobacillus rhamnosus*, LP: *Lactobacillus plantarum*, PA: *Pediococcus acidilactici*, EF: *Enterococcus faecium*, EC: *Escherichia coli*

To determine if these selected bacterial strains could influence the severity of colitis in vivo, we induced experimental colitis using DSS in mice. These bacterial strains were administered daily by oral gavage three days before and during colitis induction (Additional file 3). DSS treated mice exhibited colitis, indicated by significant weight loss, shortened colon length, elevated disease activity index (DAI, a combined score assessing weight loss, rectal bleeding and stool consistency), and colonic damage represented by colonic histological score (Fig. 1a). Notably, 3 out of 5 selected bacterial strains and the 2 positive control strains significantly alleviated colitis in mice, as indicated by markedly increased colon length, reduced DAI and colonic damages presented by H&E staining and histological score (Fig. 1b-e, Additional file 4). Although BB and EC2 reduced the histological scores of DSS-induced colitis, clinical manifestations of colitis including weight loss, shortened colon length and DAI were failed to be ameliorated (Additional file 4). And the cocktail of 5 bacterial strain (3 selective strains and 2 positive control strains) and FMT did not achieve better therapeutic effect than the single bacterial strains. Parameters like weight loss, colon length, DAI and colonic histological score showed minimal or no improvement compared with the DSS group (Additional file 5). In another more immune related colitis model induced by TNBS in mice, only LP demonstrated a protective effect on inflammation, with reduced DAI and colonic histological scores. The other strains (LR, PA, EF and EC) did not exhibit similar protective effects (Additional file 6).

### Human-derived bacterial strains reduced inflammatory responses during colitis

**Fig. 2:**
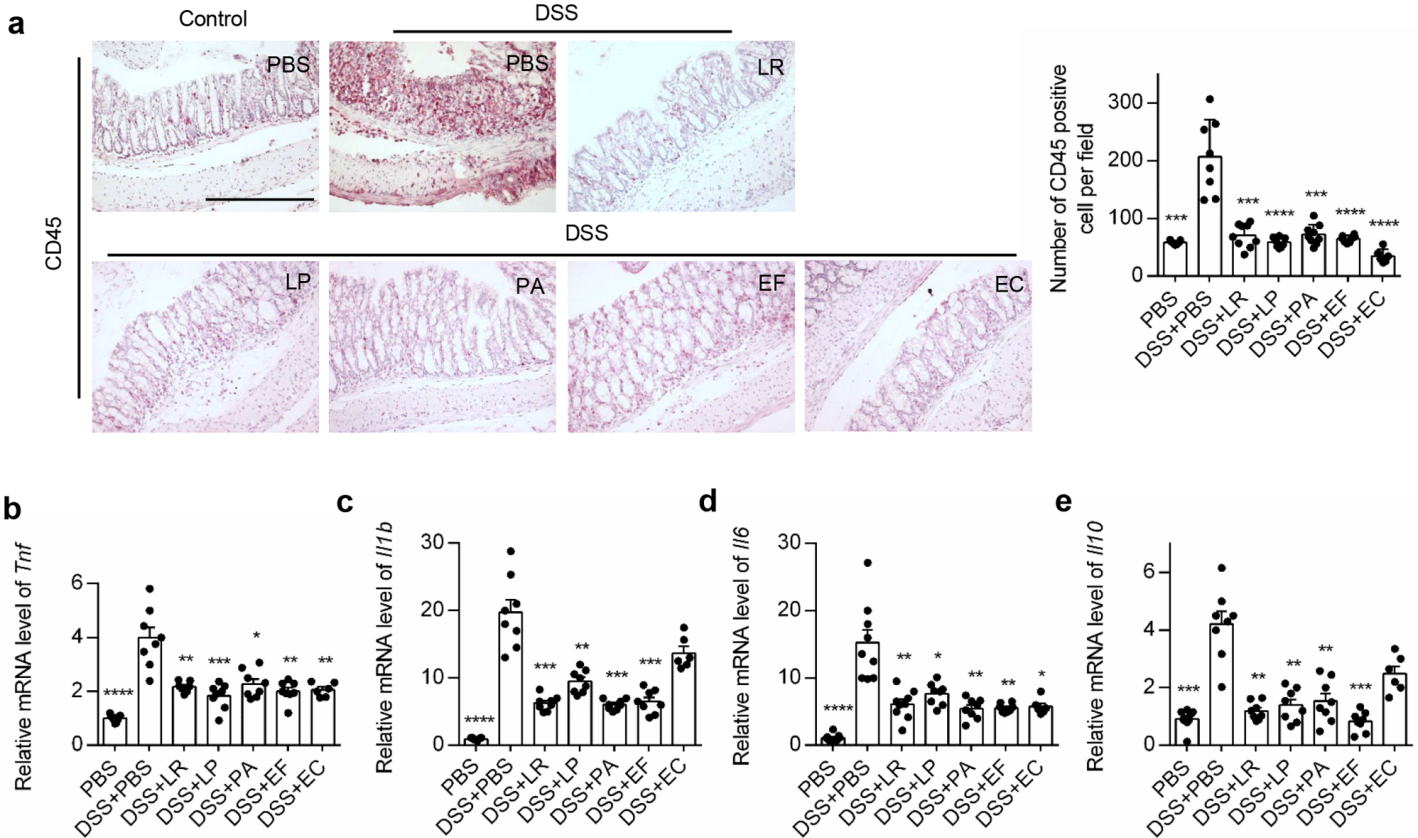
Human-derived bacteria reduced inflammatory responses in DSS-induced colitis mice. (**a**) Representative images showing immunohistological staining of CD45 in colonic tissue of colitis mice and its statistical quantification. Scale bar = 100 μm. (**b**-**e**) qPCR quantification of *Tnf* (**b**), *Il1b* (**c**), *Il6* (**d**) and *Il10* (**e**) expression in colonic tissue. *n* = 5–10 mice per group. *P < 0.05, **P < 0.01, ***P < 0.001, ****P < 0.0001 vs. DSS + PBS group by ANOVA followed by LSD post hoc test. LR: *Lactobacillus rhamnosus*, LP: *Lactobacillus plantarum*, PA: *Pediococcus acidilactici*, EF: *Enterococcus faecium*, EC: *Escherichia coli*

Since the 5 strains acted differently in two experimental mouse models, we next sought to determine how these bacterial strains effected the severity of colitis. Firstly, we investigated the inflammatory responses in mice. Infiltration of immune cells was largely reduced by all bacterial strains (2 positive strains and 3 human gut derived strains) as indicated by CD45 and F4/80 staining (Fig. 2a, Additional file 7a). The gene expression of pro-inflammatory cytokines TNF-α, IL-1β, IL-6 and IL-17a, as well as toll-like receptor 4 (TLR4) were significantly inhibited by more than 2 effective bacteria (Fig. 2b-d, Additional file 7b-c). The gene expression of IL-10, an anti-inflammatory cytokine, was also downregulated (Fig. 2e), probably indicating that the mice treated with these strains had more moderate inflammatory responses that were not strong enough to provoke the anti-inflammatory response. No significant changes of serum C-reactive protein (CRP) levels were observed in bacteria-treated mice (Additional file 7d). Moreover, serum lipopolysaccharide binding protein (LBP) remained unchanged even in DSS treated mice, indicating that DSS didn’t induce bacteremia in our study (Additional file 7e). In addition, the number of proliferative cells after colitis induction was reduced by administration of bacteria LR, LP and PA as indicated by Ki-67 staining (Additional file 7f). Collectively, these data suggest that these bacterial strains significantly mitigate a mild-inflammatory response in vivo.

### Human-derived bacterial strains enhanced intestinal barrier function

**Fig. 3:**
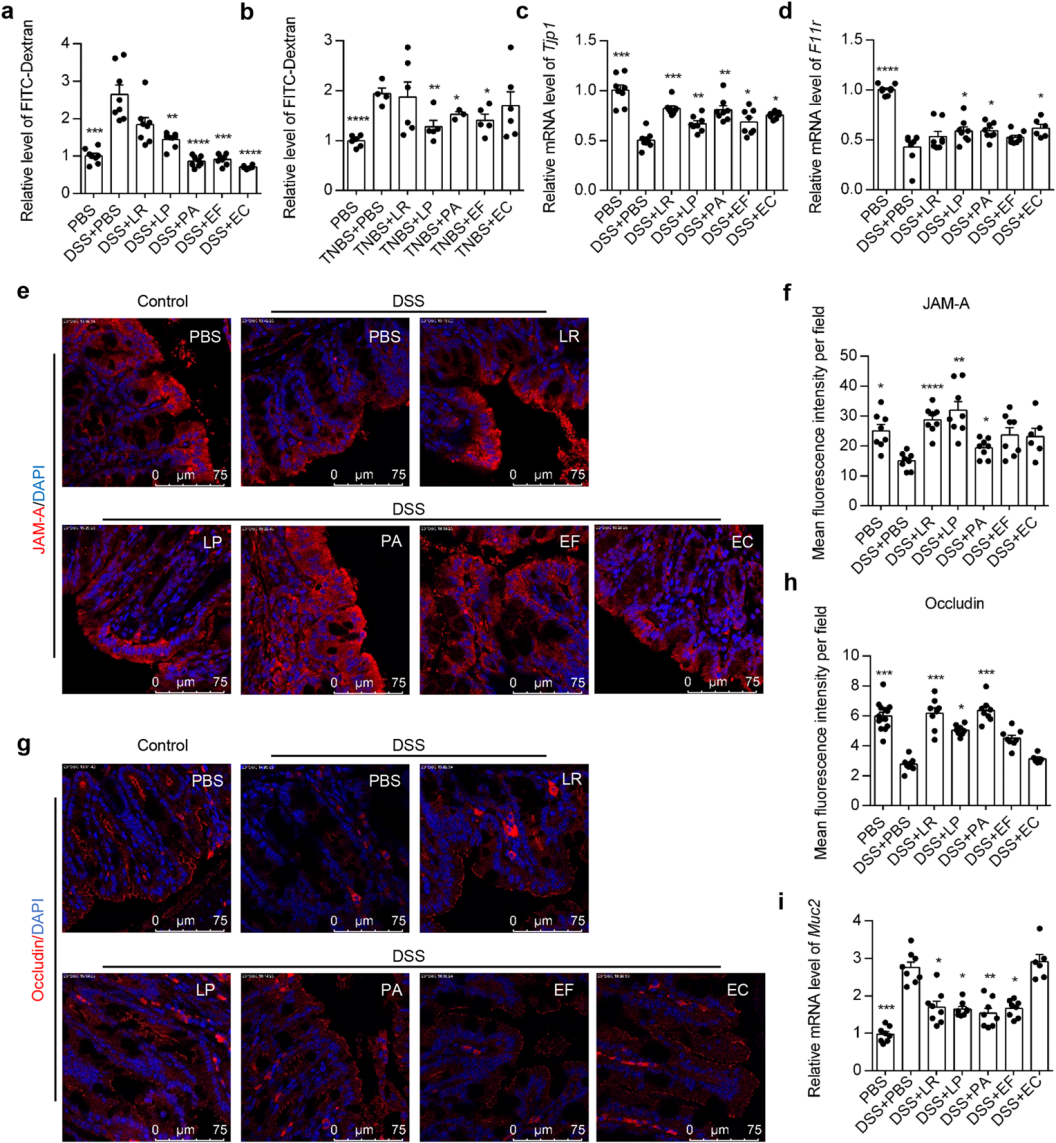
Human-derived bacteria restored gut barrier function in DSS-induced colitis model. (**a**, **b**) Relative level of FITC-dextran in serum of DSS (**a**) and TNBS (**b**) treated mice. (**c**, **d**) qPCR quantification of mRNA levels of *Tjp1* (**c**) and *F11r* (**d**) in the colonic tissue. *Gapdh* served as an endogenous control. (**e**, **f**) Representative immunofluorescence images of JAM-A (**e**) and its statistical quantification (**f**). (**g**, **h**) Representative immunofluorescence images of Occludin (**g**) and its statistical quantification (**h**). (**i**) qPCR quantification of mRNA levels of *Muc2*. *n* = 5–10 mice per group. *P < 0.05, **P < 0.01, ***P < 0.001, ****P < 0.0001 vs. DSS + PBS group by ANOVA followed by LSD post hoc test. LR: *Lactobacillus rhamnosus*, LP: *Lactobacillus plantarum*, PA: *Pediococcus acidilactici*, EF: *Enterococcus faecium*, EC: *Escherichia coli*

Since the impairment of intestinal barrier is also one of the most important signatures of colitis, we next explored whether these bacterial strains could regulate gut barrier function. Mice were gavaged with 4 kDa FITC-linked dextran, which would not be detected in the serum unless the gut barrier integrity is compromised. We found that mice treated with strains LP, PA, EF and EC but not LR exhibited significantly reduced dextran leakage in the serum compared with DSS control group, even to the same level with that of the healthy control group (Fig. 3a). LP, PA, EF also significantly reduced dextran leakage in TNBS colitis model (Fig. 3b), indicating a strong protective role of these bacterial strains on intestinal barrier function. We further detected the expression of tight junction proteins in the colon. Among the 6 kinds of tight junction we detected, the mRNA expression of JAM-A (gene name *F11r*) and ZO-1 (gene name *Tjp1*) were significantly upregulated by LP, PA and EC (Fig. 3c-d, Additional file 8). The increased expression of JAM-A in LR, LP, PA treated mice was further confirmed by immunofluorescent staining (Fig. 3e-f). Moreover, the immunofluorescent staining also showed increased occludin expression in LR, LP and PA treated mice (Fig. 3g-h). Notably, the expression of *Muc2*, the major component of mucin layer in the intestinal, was downregulated in the other bacteria groups excluded EC group (Fig. 3i), suggesting that the direct interaction between intestinal epithelial cells and bacteria maybe beneficial in enhancing the barrier function. Taken together, all these data suggest that these bacterial strains significantly enhance intestinal barrier function in vivo.

### Human-derived bacterial strains modulated the structural composition of gut microbiota

**Fig. 4:**
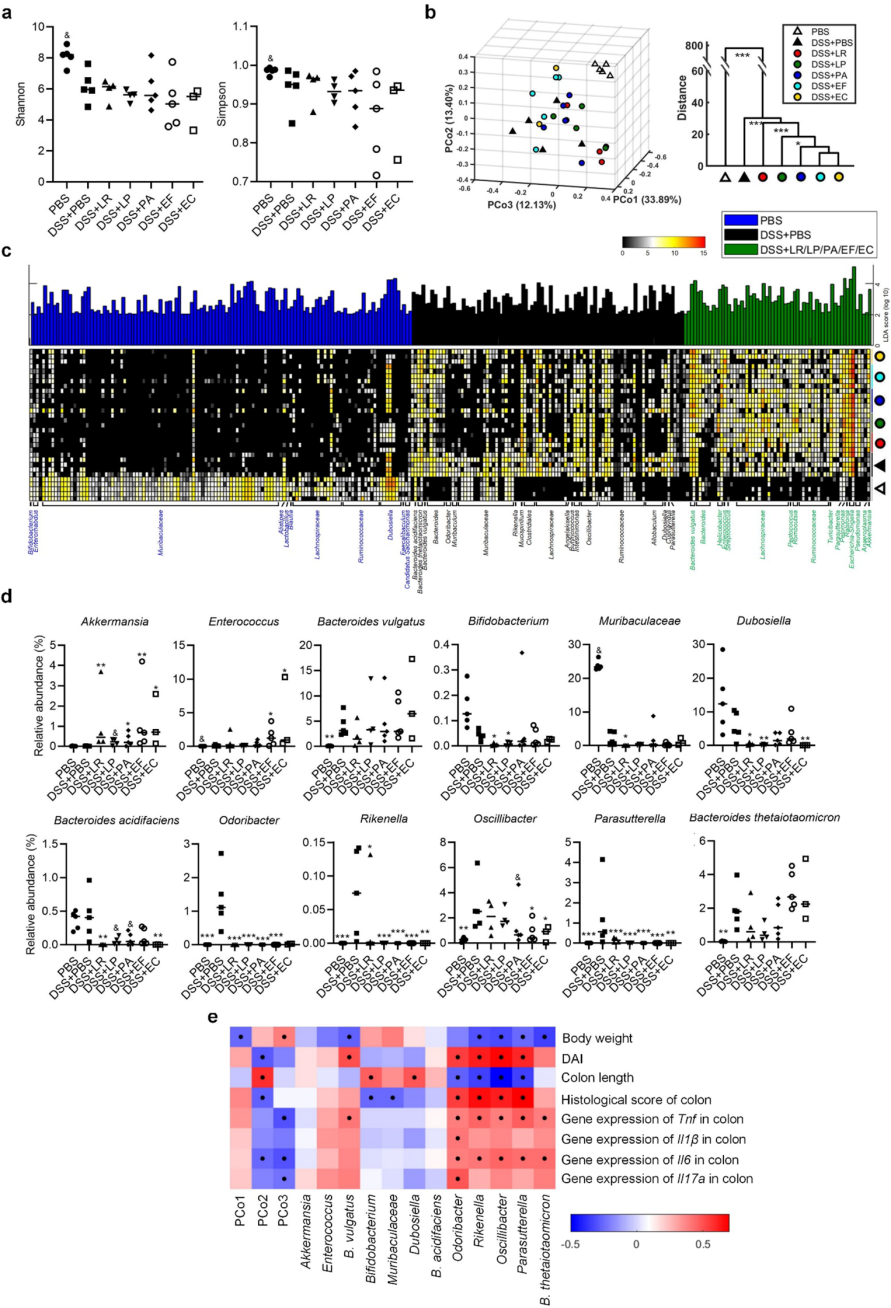
Human-derived bacteria modulated gut microbiota associated with colitis phenotype and colonic inflammation. (**a**) Shannon and Simpson diversity index. (**b**) Principal coordinate analysis (PCoA) plot based on Brey Curtis distance of ASV abundance (left) and clustering of gut microbiota based on mahalanobis distances between different groups calculated with multivariate analysis of variance test (right). (**c**) Heatmap of 269 ASV abundances in mice according to LEfSe analysis. Blue represented ASVs highest in the healthy control, black represented ASVs highest in the mice with colitis, and green represented ASVs highest in the mice with bacterial treatment. (**d**) The relative abundance of the 12 genus/species changed by the isolated bacteria. In the plot, each dot represented each mouse. The line marked the median. Differences were assessed by Mann-Whitney test. (**e**) Spearman rank correlation heatmap of parameters concerning overall severity of colitis, inflammatory cytokines and PCoA coordinates/ 12 significantly different taxa in colonic content samples. Colors red and blue denoted positive and negative association, respectively. The intensity of the colors represented the degree of associations assessed by the Spearmen’s correlations. The black dots in the blue/red cells indicated the associations were significant (*p* < 0.05). ^&^*P* < 0.1, *P < 0.05, **P < 0.01, ***P < 0.001 vs. DSS + PBS group by ANOVA followed by LSD post hoc test. *n* = 3–5 mice per group for the 16S rRNA gene sequencing of colonic content bacteria. LR: *Lactobacillus rhamnosus*, LP: *Lactobacillus plantarum*, PA: *Pediococcus acidilactici*, EF: *Enterococcus faecium*, EC: *Escherichia coli*

Since gut microbiota dysbiosis is an important feature of colitis, we next investigated what kinds of resident bacteria were modulated by these strains, and the relationship between specific modulated bacteria and different aspects of pathological features of colitis. We performed bacterial 16S rRNA gene V3-V4 region sequencing for all groups on the Illumina MiSeq platform. A total of 2,958,720 usable high-quality raw reads were obtained. The sequences were binned into 33,835 amplicon sequence variants (ASVs) at the 100% similarity level after chimeras and singleton removing. Shannon’s and Simpson’s index showed that these strains did not restore bacterial diversity decreased by DSS (Fig. 4a). Random Forest models showed the abundance of gut microbiota could be used to predict the grouping (Additional file 9a). Principal coordinate analysis (PCoA) based on Bray Curtis distances of ASV relative abundance matrix was performed to provide an overview of the gut microbiota composition of the seven groups. The PCoA scores showed that the three selective strains and the two positive control strains shifted the overall structures of the DSS-disrupted gut microbiota toward that of normal mice (Fig. 4b). Multivariate analysis of variance (MANOVA) derived from PCoA scores presented the significant separations among the healthy control, DSS, the strain groups (Fig. 4b).

Taxonomic assignment showed that there were 23 phyla, 199 families and 446 genera in the colonic content of the mice (Additional file 9b-d). At the phylum level, all the strains significantly increased the relative abundance of *Verrucomicrobia* (Additional file 9b). At the family level, all the strains increased bacteria belonging to *Peptostreptococcaceae* and *Akkermansiaceae* (Additional file 9c). At the genus level, at least two of the three human gut derived strains increased the relative abundances of *Romboutsia*, *Faecalibaculum*, and *Akkermansia*, and decreased that of *Odoribacter* and *Clostridium sensu stricto* 1 (Additional file 9d).

Then a linear discriminant analysis (LDA) effect size (LEfSe) was employed to identify specific bacterial phylotypes that were different among the healthy group (PBS), colitis group (DSS + PBS) and the treatment group (DSS + LR/LP/PA/EF/EC). There were 269 different ASVs among the three groups, with 122 ASVs enriched in the healthy group, 87 ASVs in the colitis group and 60 ASVs in the treatment group (Fig. 4c). After combining the ASVs from the same genus or species, we got 12 significantly different genus/species. Among them, two kinds of bacteria including *Akkermansia* and *Enterococus* were enriched; while, seven bacteria including *Bifidobacterium, Dubosiella, Bacterioides acidifaciens, Odoribacter, Rikenella, Oscillibacter*, and *Parasutterella* were reduced by at least two of the strains (Fig. 4d). Next, Spearman’s correlation analysis was used to determine associations between colitis/inflammation parameters and overall microbiota composition represented by the PCoA coordinates/ twelve specific symbiotic bacteria which were modulated by the strains (Fig. 4e). Compositional changes of the gut microbiota along PCo2 and PCo3 were significantly negatively associated with colitis and gut inflammation (Fig. 4e). More specifically, symbiotic *Odoribacter, Rikenella, Oscillibacter*, and *Parasutterella* were significantly positively correlated with colitis and gut inflammation (Fig. 4e). All these results suggest that these bacterial strains reduce inflammation-related bacteria *Odoribacter*, *Rikenella*, *Oscillibacter* and *Parasutterella*.

**Fig. 5:**
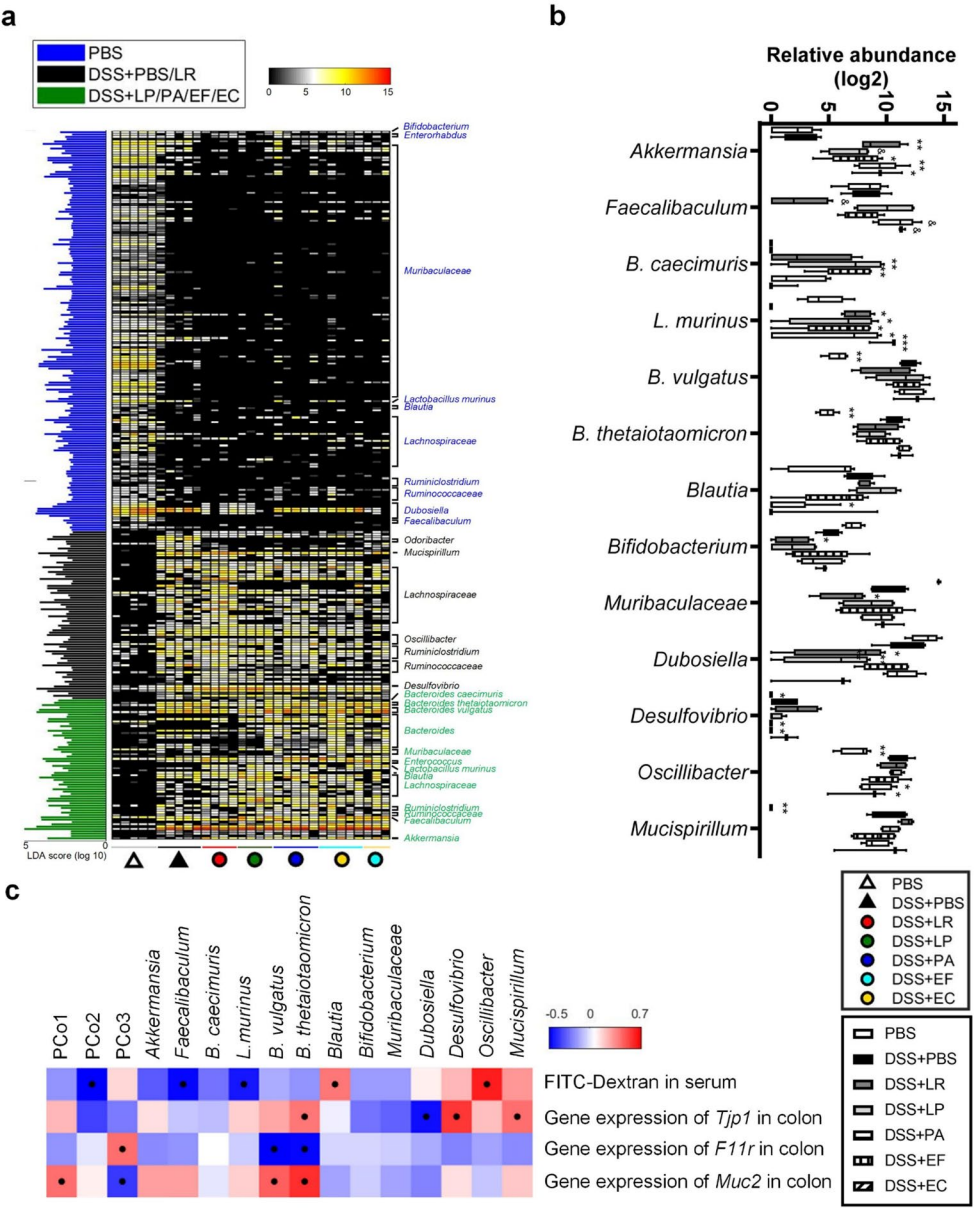
Human-derived bacteria modulated gut microbiota associated with gut barrier. (**a**) Heatmap of 303 ASV abundances in mice according to LEfSe analysis. Blue represented ASVs highest in the healthy control, black represented ASVs highest in the mice with gut dysfunction, and green represented ASVs highest in the mice with good gut barrier under the help of the bacterial strains. (**b**) The relative abundance of the 13 genus/species changed by the isolated bacteria. In the box plot, the bottom and top were the 25th and 75th percentile, respectively. A line within the box marked the median. Whiskers above and below the box indicated the maximum and minimum values. Differences were assessed by Mann-Whitney test. (**c**) Spearman rank correlation heatmap of gut barrier parameters and PCoA coordinates/ 13 significantly different taxa in colonic content samples. Colors red and blue denoted positive and negative association, respectively. The intensity of the colors represented the degree of associations assessed by the Spearmen’s correlations. The black dots in the blue/red cells indicated the associations were significant (*p* < 0.05). ^&^*P* < 0.1, *P < 0.05, **P < 0.01, ***P < 0.001 vs. DSS + PBS group by ANOVA followed by LSD post hoc test. *n* = 3–5 mice per group for the 16S rRNA gene sequencing of colonic content bacteria. LR: *Lactobacillus rhamnosus*, LP: *Lactobacillus plantarum*, PA: *Pediococcus acidilactici*, EF: *Enterococcus faecium*, EC: *Escherichia coli*

Given that LR mitigated colitis, increased the expression of tight junction proteins, but still failed to restore gut barrier as indicated by dextran leakage, we assume that this phenomenon might be related to resident gut microbiota. We combined DSS group and LR group as the “compromised gut barrier group”, and LP group and the three isolated strain groups as the “restored gut barrier group”. (Fig. 5a). LEfSe was performed to identify specific bacterial phylotypes for which the abundance was different among the healthy control group (PBS), compromised gut barrier group (DSS + PBS/LR) and restored gut barrier group (DSS + LP/PA/EF/EC). There were 303 ASVs different among the three groups, with 171 ASVs enriched in the healthy control group, 72 ASVs enriched in the compromised gut barrier group, and 60 ASVs enriched in the restored gut barrier group (Fig. 5a). After combining the ASVs from the same genus or species, we got 13 significantly different genus/species, which were divided into five groups according to their abundance in each group (Fig. 5b). The first group of bacteria included *Akkermansia*, *Faecalibaculum*, and *Bacteroides caecimuris*. The three kinds of bacteria were at the same level in PBS and DSS groups, and increased by at least two of the selected strains. Notably, LR decreased the relative abundance of *Faecalibaculum* (Fig. 5b). The second group was *Lactobacillus murinus*, which was lower in DSS group but higher in all the groups administrated with selected bacteria (Fig. 5b). The third group included *Bifidobacterium*, *Muribaculaceae*, and *Dubosiella*, which were slightly reduced in the DSS group and significantly lower in the LR group (Fig. 5b). The fourth group had *Desulfovibrio*, *Oscillibacter*, and *Mucispirillum*, which were enriched by DSS but reduced by LP and three selected strains (Fig. 5b). The fifth group included *B. vulgatus*, *B. thetaiotaomicron*, and *Blautia*, which were enriched in the DSS group and bacteria groups (Fig. 5b). We next used Spearman’s correlation analysis to determine associations between gut barrier parameters and overall microbiota composition/ thirteen modulated bacteria (Fig. 5c). Bacteria along PCo2 were significantly associated with FITC levels tested in the serum (Fig. 5c). At the genus/species level, symbiotic *Faecalibaculum* and *L. murinus* were significantly negatively correlated with gut permeability, while *Oscillibacter* was significantly positively correlated with gut permeability (Fig. 5c). All these results indicate that the human gut derived bacterial strains have the potential to enrich gut barrier-related bacteria *Faecalibaculum* and *L. murinus*.

### Human-derived bacterial strains influenced SCFAs metabolism

**Fig. 6:**
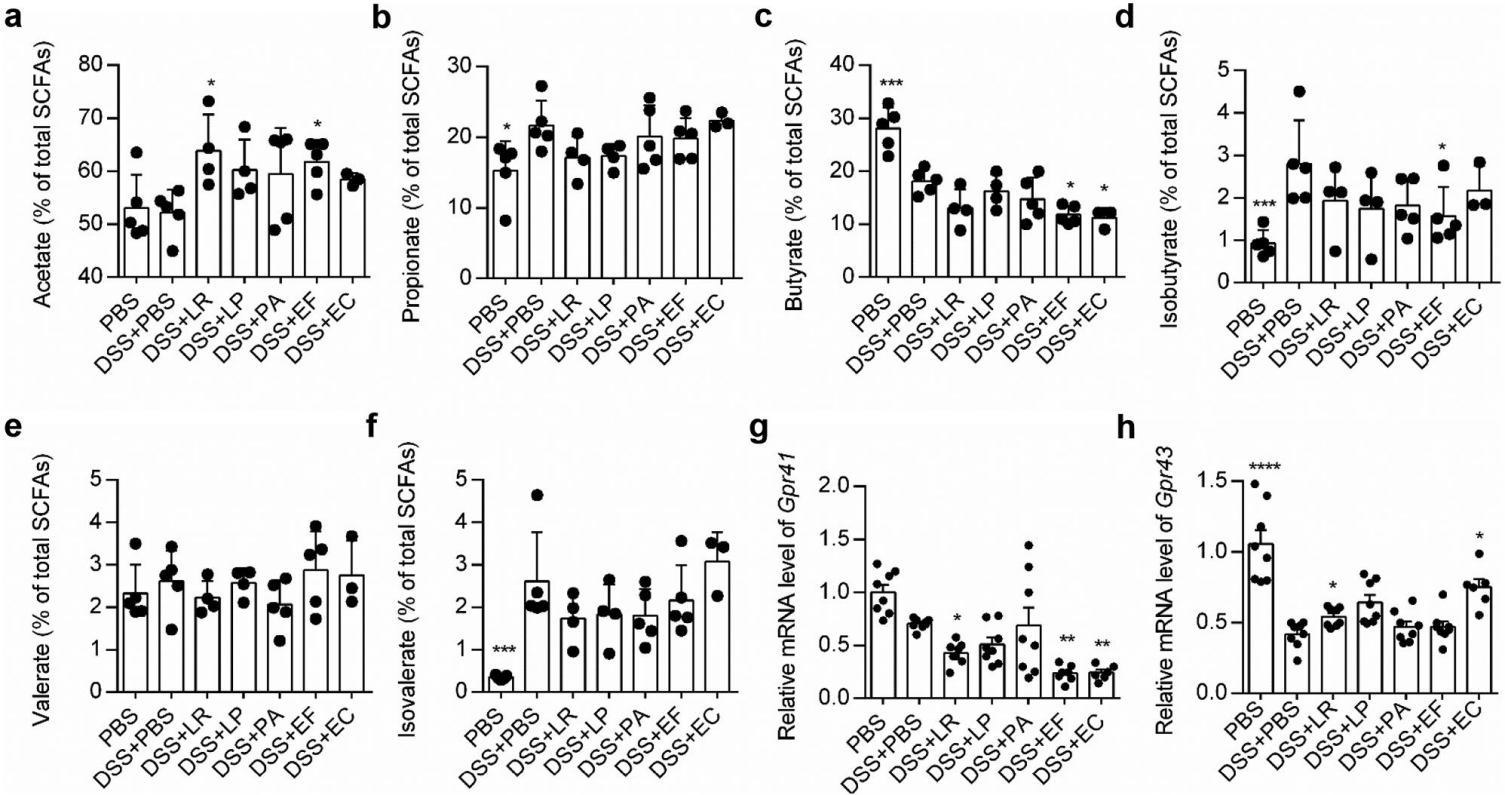
Human-derived bacteria changed metabolites composition in cecum content samples from DSS-induced colitis model. (**a**-**f**) Relative abundance of acetate (**a**), propionate (**b**), butyrate (**c**), isobutyrate (**d**), valerate (**e**) and isovalerate (**f**) quantified by GC-MS. (**g**, **h**) qPCR quantification of *Gpr41* (**g**) and *Gpr43* (**h**) in the colonic tissue. *Gapdh* served as an endogenous control. *n* = 5–10 mice per group. ^&^*P* < 0.1, *P < 0.05, **P < 0.01, ***P < 0.001, ****P < 0.0001 vs. DSS + PBS group by ANOVA followed by LSD post hoc test. LR: *Lactobacillus rhamnosus*, LP: *Lactobacillus plantarum*, PA: *Pediococcus acidilactici*, EF: *Enterococcus faecium*, EC: *Escherichia coli*

SCFAs are the end products of dietary fibers fermented by gut microbiota, and are important mediators for the treatment of bowel inflammatory diseases. We further analyzed SCFA levels in each group (Fig. 6a-f). Among all the SCFAs, only the relative content of acetate was increased in mice treated by LR, LP, PA and EF (Fig. 6a). In contrast, the relative contents of butyrate and isobutyrate were reduced by LR, EF and/or EC (Fig. 6c-d). In consistence with changes in SCFAs, the SCFA receptors also exhibited significant changes. The expression of G-protein-coupled receptor 41 (GPR41), which is most sensitive to butyrate and propionate, was downregulated by LR, EF and EC (Fig. 6g), while the gene expression of GPR43, which is most sensitive to acetate was significantly increased by LR and EC (Fig. 6h). All these suggest that the strains can improve the acetate metabolism, and this might participate in the protective effect of these bacterial strains.

In addition, we also detected the gene expression levels of several other gut microbial metabolites-related receptors including the aryl hydrocarbon receptor (AHR), bile acid receptor TGR5 and farnesoid X receptor (FXR) (Additional file 10). We found that the mRNA level of TGR5 and FXR were markedly decreased by LP, EF and EC (Additional file 10b, c), indicating that bile acid metabolism and its related metabolites might also participate in the protective effect of these bacterial strains, which requires further exploration.

### The protective role of these bacterial strains was dependent on the preexisting gut microbiota

**Fig. 7:**
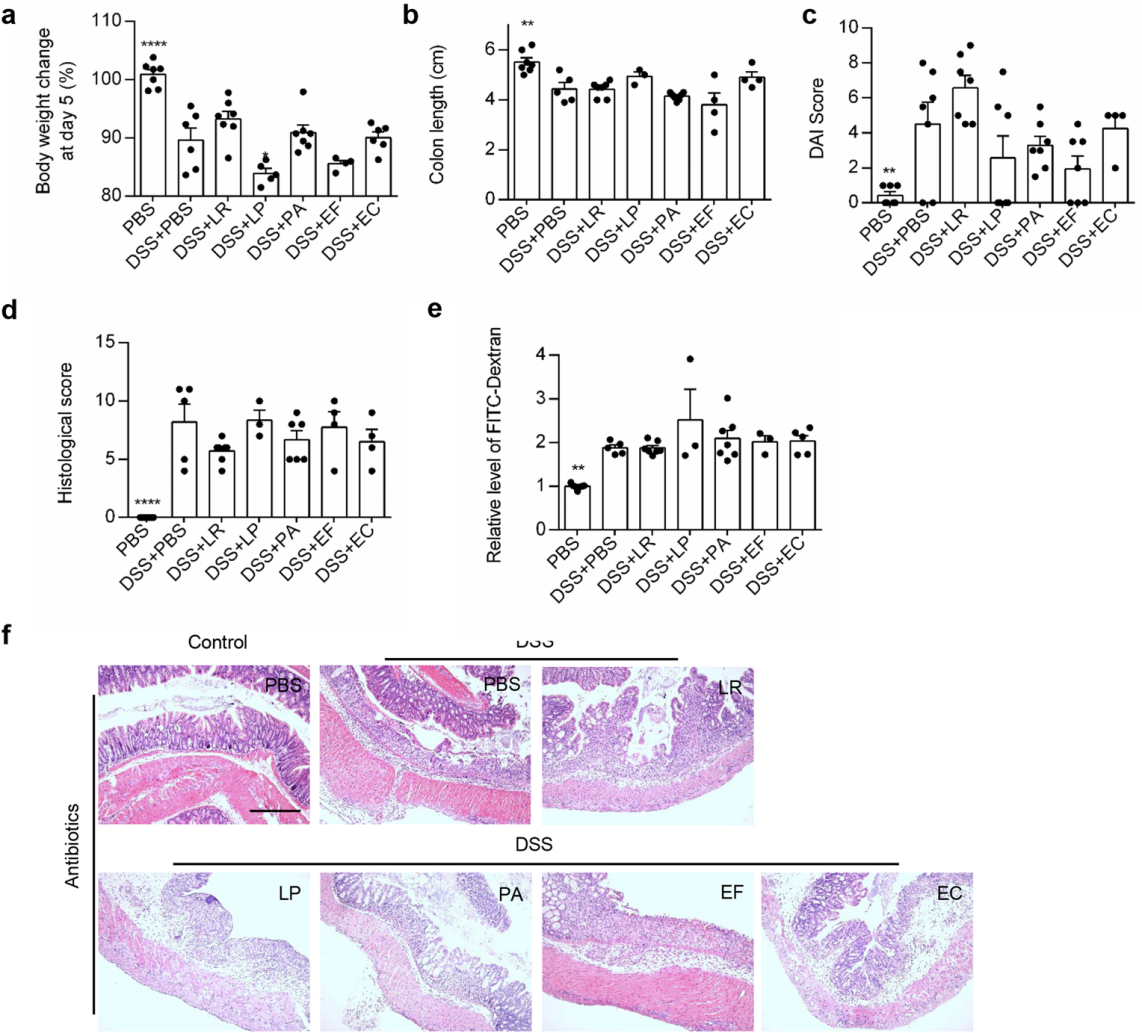
Ablation of preexisting gut microbiota abolished the protective role of specific bacterial strains. (**a**) Percentage of body weight changes at day 5 after DSS administration. (**b**) Colon length of mice at day 5 after DSS administration. (**c**) Quantification of the DAI score of mice at day 5 after DSS administration. (**d**) Quantification of histological scores of colon tissue. (**e**) Representative images showing H&E staining of colonic tissue in DSS-induced colitis mice. Scale bar = 100 μm. (**f**) Relative level of FITC-Dextran in serum of DSS-treated mice. *n* = 5–10 mice per group. **P < 0.01, ****P < 0.0001 vs. DSS + PBS group by ANOVA followed by LSD post hoc test. LR: *Lactobacillus rhamnosus*, LP: *Lactobacillus plantarum*, PA: *Pediococcus acidilactici*, EF: *Enterococcus faecium*, EC: *Escherichia coli*

Since the endogenous gut microbiota and its metabolites were significantly influenced by these bacterial strains and correlated with colitis, we next determined if these bacterial strains exerted their protective effects on colitis through the endogenous gut microbiota. Mice were treated with broad-spectrum antibiotics for 2 weeks to ablate preexisting bacteria as previously described (Additional file 3) [27]. We found that the protective effects of these bacteria on colitis phenotype and gut barrier function were totally abolished in antibiotic-treated mice, as indicated by unimproved weight loss, DAI score, colon length, histological score of H&E staining, and serum FITC-dextran level (Fig. 7A–F). These results suggest that these bacterial strains exert their protective role in vivo through preexisting microbiota.

## Discussion

Despite the fact that the association between colitis and the imbalance of gut microbiota is undisputable, the role and the mechanisms of the endogenous gut microbiota in ameliorating colitis remain elusive. Here, our study showed the indispensable function of the endogenous gut microbiota. The new findings of our study compared with previous related studies are: (1) The selected human gut derived bacterial strains *P. acidilactici*, *E. faecium*, and *E. coli* significantly mitigated colitis and repaired gut barrier function, while ablation of resident gut microbiota using broad-spectrum antibiotics fully abolished protective effects on colitis and gut barrier function of these isolated strains; (2) All the three selective strains increased the relative abundance of endogenous bacteria belonging to *Faecalibaculum* and *L. murinus*, which were significantly positively correlated with gut barrier; and reduced the relative abundance of the endogenous bacteria from *Odoribacter*, *Rikenella*, *Oscillibacter* and *Parasutterella*, which were positively correlated with inflammation; (3) Strain PA and EF increased the relative level of gut microbiota metabolite acetate, and strain EC promoted the mRNA expression of acetate receptor GPR43. All these suggest that the composition and metabolism of endogenous gut microbiota are required for human gut bacteria-mediated amelioration of colitis.

As demonstrated in our study, the selected bacterial strains protected mice from experimental colitis through restoring the homeostasis of gut microbiota, while antibiotics completely eliminated the therapeutic effect by removing the gut microbiota, emphasizing the importance of a balanced gut microbiota in the treatment of colitis. Although antibiotics were sometimes used for the treatment of acute severe ulcerative colitis [28], their effects in colitis remain controversial [29]. In consistence with our results, application of antibiotics was even reported to aggravate colitis by inducing gut dysbiosis, loss of immune tolerance [30] and impairment of gut barrier [31, 32]. In addition, probiotics induced a markedly delayed and persistently incomplete post-antibiotic reconstitution of resident gut microbiota [33]. All these remind us that antibiotics should be prudently used before clinical application of probiotics because the resident gut microbiota might be the target of probiotics.

Generally, to ameliorate the symptoms of IBD, probiotics bacterial strains are isolated from diet or the feces of healthy adult donors, and fecal microbial suspension for FMT are also produced from the fecal samples of healthy donors to minimize risks of disease transmission [13]. However, here we isolated bacterial strains from the feces of a healthy infant and a patient with colitis in remission, and amazingly, they markedly repaired intestinal barrier dysfunction and significantly reduced inflammation in mouse colitis models. Infants have the developing gut, with high permeability, simple and immature gut microbiota and imperfect immune system. Therefore, the bacteria in it adapt to this environment, and may have the function of keeping gut barrier intact and healthy and not over-activating immune response, and finally maintaining the healthy steady state of the host’s gut. In contrast, patients with colitis have the gut recovering from severe damages. The bacteria in the intestinal tract may have the function of restoring the gut barrier or stimulating the activation of the protective mechanism of the gut. Bacteria from developing guts (infants) or recovering guts (colitis patients in remission) may have unique protective properties. Specifically, these bacteria may help maintain intestinal barrier integrity and avoid excessive immune activation, thereby contributing to gut health. Although the microbiota of mice is very different from human, often human bacterial strains do not even colonize mice [34], and the abundances of certain bacteria vary greatly between mice and human, such as *Lactobacillus*, *Faecalibacterium* [35], our isolated bacterial strains from human volunteers were effective in attenuating gut barrier dysfunction and colitis in mice.

In our study, the three selective strains didn’t increase the bacterial diversity, but induced partial and significant changes in overall gut microbiota structure disrupted by DSS. The overall structural changes of gut microbiota (PCos of the PCoA plot) were associated with colitis phenotypes in the mouse model. It is thus important to identify changes in species-level or subspecies-level phenotypes in response to the treatment of these strains. Although it showed strain specificity in modulation of gut microbiota by probiotics, the three strains here shared some common features in regulating gut microbiota composition, particularly in increasing the relative abundance of beneficial bactria *Faecalibaculum* and *L. murinus* and decreasing that of inflammation-related bacteria *Odoribacter*, *Rikenella*, *Oscillibacter* and *Parasutterella*. Notably, increased *Faecalibaculum* and *L. murinus* were significantly negatively correlated with gut permeability in this study. In previous studies, *L.murinus* was shown to reduce intestinal barrier damage in rat with necrotizing enterocolitis [36], and decrease the intestinal permeability and serum endotoxin load in gnotobiotic mice colonized with the gut microbiota from old mice [37]. Similarly, *Faecalibaculum* could be raised by the probiotics [38] and *F. rodentium* from this genus protected from colonic tumor growth in mice [39]. However, the specific protective role of *Faecalibaculum* on the gut barrier remains to be elucidated. While, decreased *Odoribacter*, *Rikenella*, *Oscillibacter* and *Parasutterella* were positively correlated with inflammation here. In some researchers’ studies, *Odoribacter*, an isovaleric acid-producing bacteria, has been isolated from the inflammatory habitat [40] and suggested to have proinflammatory properties [41]. While, in some other researches, *Odoribacter*, was associated with anti-inflammatory properties and found to be correlated with the positive clinical response of UC patients to FMT therapy [42, 43]. These contradictory results indicate that further functional experiments are needed to verify the results of these correlation studies. Consistent with our results, probiotic cocktail VSL#3 decreased *Oscillibacter* in colitis-associated carcinogenesis mouse model [44], and *Oscillibacter* could cause bacteremia [45]. *Parasutterella*, a new member of the core gut microbiota [46], was recently reported to be associated with intestinal chronic inflammation [47]. All these previous studies support that we here identify the modulated bacteria which had the potential to enhance gut barrier function or induce inflammation. The modulation of gut microbiota represented by increase of bacteria enhancing gut barrier and reduce of proinflammatory bacteria may be an important mechanism for the treatment of colitis by the selective bacteria.

Short chain fatty acids (SCFAs), which are produced by fermentation of dietary fiber by intestinal microbiota, are considered as a critical element to protect against the development of intestinal inflammation [22]. A feature of patients with ulcerative colitis or other colitis diseases is the reduction in SCFAs, including acetate, propionate and butyrate [48], and decreased SCFA-producing bacteria, such as *Clostridium* [49]. Moreover, increased intake of fermentable dietary fiber or SCFAs, is clinically beneficial in the treatment of colitis [50]. Acetate, the most abundant SCFAs, binds the GPR43 [22]. *Gpr43*-deficient mice presented with exacerbated inflammation in colitis models, and germ-free mice, which are devoid of bacteria and express little or no SCFAs, showed a similar dysregulation of inflammatory responses [22], suggesting that SCFAs-GPR43 interactions may affect inflammation. SCFAs could reduce inflammation by mediating the homeostasis of Treg cells and T cell response during colitis [23, 51]. In our study, we also noticed that the relative contents of acetate or expression of receptor GPR43 were increased in the three strains treated group. Moreover, The increased *L.murinus* and *Faecalibaculum* were reported to produce acetate [52, 53]. Together, these observations suggest that the gut microbial acetate metabolism provides a link between the gut microbiota and host inflammatory responses.

## Conclusions

To summarize, our study identified three human-derived bacterial strains that exerted protective effects on colitis through the modulation of endogenous gut microbiota in mouse colitis models. Bacterial acetate metabolism might be involved in this effect, but further experiments are warranted to elucidate the underlying mechanisms. Although translation of these findings to clinical applications remains a significant challenge, ‘gut microbiota-targeted’ bacterial strain intervention strategies may become an important element in the amelioration of colitis.

## Methods

### Human donor

Two donors were selected for this study. Donor 1 was a healthy, breast-fed male infant aged four and a half months, who had been fed on supplementary food half a month prior. Donor 2 was a 48-year-old male patient diagnosed with ulcerative colitis, currently in remission following treatment with hormone and 5-aminosalicylic acid. His colonoscopy revealed no ulcers or purulent secretions in the gut membrane. Written informed consent was obtained from the patient and the guardian of the infant. All studies involving human samples were approved by the ethics committee of Shanghai General Hospital (2019SQ157) and performed in accordance with the Declaration of Helsinki.

### Fecal suspension and bacterial isolation

Fresh fecal samples of the donors were collected in an oxygen-free environment and quickly transferred to an anaerobic workstation. There they were homogenized and diluted 5-fold with sterile, pre-reduced 0.01 M phosphate buffer saline (PBS, pH 7.4). Any food derived debris was removed through sterile gauze. For bacterial isolation, the fecal samples were anaerobically incubated with de Man, Rogosa, Sharpe (MRS) for 48 h, and aerobically incubated with Luria–Bertani (LB) mediums for 24 h. After marking every single colony twice, the bacterial strains were preserved for further usage.

### Animal trial

All procedures were approved by the Animal Ethics Committee of Shanghai General Hospital (2019-A053-01), and the research methods were carried out by the approved guidelines. C57BL/6 mice (6–8 weeks, male) were purchased from Shanghai SLAC Laboratory Animal Co Ltd. Mouse colitis models were induced by DSS (MP, #216,011,050) or TNBS (Sigma, #P2297). The DSS colitis model was induced by adding 4% DSS in drinking water for 5 days. Mice were sacrificed at day 5. The TNBS colitis model was induced by administering 150 mg/kg TNBS in 50% ethanol intracolonically. Mice were sacrificed at day 3. Antibiotic pre-treatment was performed by adding ampicillin (1 mg/mL, MCE, #HY-B0522), gentamicin (1 mg/mL, MCE, #HY-A0276), metronidazole (1 mg/mL, MCE, #HY-B0318), neomycin (1 mg/mL, MCE, #HY-B0470) and vancomycin (0.5 mg/mL, MCE, #HY-B0671) to autoclaved drinking water, with 2% sucrose used as the sweetener as previously described [54]. Mice were fed with antibiotic-containing water for 2 weeks. Twenty-four hours after changed into normal autoclaved water, mice were further induced with colitis and treated with bacterial strains. Isolated strains and fecal bacterial suspension of donor 1 were intragastric administrated to mice. *Lactobacillus reuteri* (LR) and *L. plantarum* (LP) served as positive controls based on the previous studies in which they could protect mice from DSS-induced colitis [55, 56]. LR, LP, PA, EF and BB were cultured in MRS medium to the plateau period, and EC and EC2 were in LB medium to the plateau period. A dose of 10^9^ colony-forming units (CFUs) of each bacterial strain was resuspended in 100ul PBS (or fecal suspension) and administered to mice daily by oral gavage three days before colitis induction. Mice treated with equal volume of PBS served as the blank control. During the final sample collection, mice were anesthetized using 1% pentobarbital sodium (50 mg/kg) during the whole process and after sample collection, mice were sacrificed using cervical dislocation as the euthanasia method.

### Gut permeability measurement

Mice were starved overnight and administered with fluorescein isothiocyanate (FITC)-dextran (250 mg/kg, Sigma, #FD40) through oral gavage. Four hours after the administration, mice were sacrificed and blood samples were collected. The concentration of serum FITC-dextran was determined using a fluorescent microplate reader, with excitation at 485 nm and measurement at 519 nm.

### Hematoxylin and eosin (H&E), immunofluorescence, and immunohistochemistry staining (IHC)

Fresh tissues were fixed with 4% neutral paraformaldehyde at room temperature for 12 to 24 h, embedded in paraffin and processed into 4 μm sections. H&E staining was done as previously described [57]. Antigen retrieval of immunofluorescence staining was achieved with heat in citrate antigen retrieval solution (Sangon, #E673002). The sections was then incubated with JAM-A antibody (Abcam, #ab180821) or Occludin (Invitrogen, #33-1500) at a 1:100 dilution overnight at 4℃ and then incubated with Alexa Fluor® 594 Conjugated secondary antibody (CST, #8889). DAPI was used for nucleus staining. Sections were imaged with fluorescent microscope (LEICA). Mean fluorescence intensity were quantified using the LAS X software.

Antigen retrieval of IHC staining was achieved by 10 min digestion with proteinase K (20 µg/ml, Beyotime, #ST532). The sections were then incubated with CD45 (CST, #55,307) or F4/80 (Abcam, #ab6640) antibody at a 1:100 dilution overnight at 4℃ and then incubated with alkaline phosphatase-linked secondary antibody (Abcam, #ab6846). SIGMA FAST™ Fast Red TR/Naphthol AS-MX Tablets (Sigma-Aldrich, #F4648) was used for the detection.

### Assessment of histological score and disease activity index (DAI)

Histological score was determined in H&E sections. The severity of inflammation (0 = none, 1 = mild, 2 = moderate, and 3 = severe), the level of inflammation involvement (0 = none, 1 = mucosa, 2 = mucosa and submucosa, and 3 = transmural), and the extent of epithelial/crypt damage (0 = none, 1 = basal 1/3, 2 = basal 2/3, 3 = crypt loss, 4 = crypt and surface epithelial destruction) were assessed and summed up to one histological score. All sections were analyzed by two experienced pathologists independently. The DAI score was evaluated by monitoring body weight loss (0 = 0%, 1 = 1–5%, 2 = 5–10%, 3 = 10–20%, 4 = >20%), stool consistency (0 = normal, 1 = loose, 2 = diarrhea) and rectal bleeding (0 = negative, 1 = +, 2 = ++, 3 = +++, 4 = gross bleeding).

### Quantification of host gene expression

Total RNA was extracted using the TRIzol reagent (Invitrogen, #15,596,026) and reverse transcribed into cDNA using the PrimeScript™ RT reagent Kit (Takara, #RR037A) according to the manufacturer’s instructions. Quantitative PCR (qPCR) was performed using TB Green® Premix Ex Taq™ (Takara, #RR420A). Each qPCR reaction was performed in triplicate, and the gene expression levels were determined by the 2^−ΔΔCt^ algorithm. The primers are listed as follows: *Il1b* forward: 5’-TTGACGGACCCCAAAAGAT-3’, reverse: 5’-GAAGCTGGATGCTCTCATCTG-3’; *Il6* forward: 5’-TTCATTCTCTTTGCTCTTGAATTAGA-3’, reverse: 5’-GTCTGACCTTTAGCTTCAAATCCT-3’; *Tnf* forward: 5’-TCTCTTCAAGGGACAAGGCTG-3’, reverse: 5’-ATAGCAAATCGGCTGACGGT-3’; *Il10* forward: 5’-GGTTGCCAAGCCTTATCGGA-3’, reverse: 5’-ACCTGCTCCACTGCCTTGCT-3’; *Il17a* forward: 5’-CAGGGAGAGCTTCATCTGTGT-3’, reverse: 5’-GCTGAGCTTTGAGGGATGAT-3’; *Il17f* forward: 5’-CCCAGGAAGACATACTCAGAAGAAAA-3’, reverse: 5’-GCAAGTCCCAACATCAACAG-3’; *Tjp1* forward: 5’-GCAAGTCCCAACATCAACAG-3’, reverse: 5’-GCAATGGTGGTCCTTCACCT-3’; *Cldn1* forward: 5’-CTGGCTTCGCTGGGATGGAT-3’, reverse: 5’-TATCTGCCCGGTGCTTTGCG-3’; *Cldn2* forward: 5’-GTCATCGCCCATCAGAAGAT-3’, reverse: 5’-ACTGTTGGACAGGGAACCAG-3’; *Cldn5* forward: 5’-GCTCTCAGAGTCCGTTGACC-3’, reverse: 5’-CTGCCCTTTCAGGTTAGCAG-3’; *Ocln* forward: 5’-CACACTTGCTTGGGACAGAG-3’, reverse: 5’-TAGCCATAGCCTCCATAGCC-3’; *Muc2* forward: 5’-GATGGCACCTACCTCGTTGT-3’, reverse: 5’-GTCCTGGCACTTGTTGGAAT-3’; *Tlr4* forward: 5’-AGGTTGAGAAGTCCCTGCTG-3’, reverse: 5’-CGAGGCTTTTCCATCCAATA-3’; *Ahr* forward: 5’-AGACCGGCTGAACACAGAGT-3’, reverse: 5’-GTCAGCAGGGGTGGACTTTA-3’; *Gpr43* forward: 5’-GGCTTCTACAGCAGCATCTA-3’, reverse: 5’-AAGCACACCAGGAAATTAAG-3’; *Gpr41* forward: 5’-GTGACCATGGGGACAAGCTTC-3’, reverse: 5’-CCCTGGCTGTAGGTTGCATT-3’; *Fxr* forward: 5’-GCAACCAGTCATGTACAGATTC-3’, reverse: 5’-TTATTGAAAATCTCCGCCGAAC-3’; *Tgr5* forward: 5’-GCCTCATCGTCATCGCCAACC-3’, reverse: 5’-GGAAGAAGCAGCCAGCAGGTG-3’; *Gapdh* forward: 5’-CAAGGCTGTGGGCAAGGTCATC-3’, reverse: 5’-TCTCCAGGCGGCACGTCAG-3’.

### 16S rRNA gene sequencing

Colonic content samples were collected from the mice after the sacrifice. Bacterial genomic DNA was extracted from colonic content samples using bead beating and the OMEGA Soil DNA Kit (Omega Bio-Tek, #M5635-02) following the manufacturer’s instructions. The extracted DNA was used as the template to amplify the V3-V4 region of the 16S rRNA gene by using the forward primer 338 F (5’-ACTCCTACGGGAGGCAGCA-3’) and the reverse primer 806R (5’-GGACTACHVGGGTWTCTAAT-3’). Sample-specific 7 bp barcodes were incorporated into the primers for multiplex sequencing. PCR amplicons were purified with Vazyme VAHTSTM DNA Clean Beads (Vazyme, #N411-03) and quantified using the Quant-iT PicoGreen dsDNA Assay Kit (Invitrogen, #P7589). Amplicons were pooled in equal amounts, and pair-end 2 × 250 bp sequencing was performed using the Illumina Miseq platform with MiSeq Reagent Kit v3 (Illumina, MS-102-3003).

### Bioinformatics and statistical analysis of 16S rRNA gene sequencing data

The microbiome bioinformatics pipeline was performed with QIIME2 2019.4 [58] and R packages (v3.2.0). Briefly, raw sequence data were demultiplexed using the demux plugin following by primers cutting with cutadapt plugin. Sequences were then quality filtered, denoised, merged and chimera removed using the DADA2 plugin. Non-singleton amplicon sequence variants (ASVs) were aligned with mafft and used to construct a phylogeny with fasttree2. Sequences were rarefied to 40,214 sequences per sample. Taxonomy was assigned to ASVs using the classify-sklearn naïve Bayes taxonomy classifier in feature-classifier plugin against the SILVA Release 132 Database. Alpha-diversity metrics (Shannon and Simpson) were estimated using the diversity plugin. Random forest analysis was performed using QIIME2 with default settings. After beta-diversity metrics based on Bray-Curtis dissimilarity were calculated, the significance of differentiation in microbiota structure among groups was assessed by multivariate analysis of variance (MANOVA) test in MATLAB R2018b. Linear discriminant analysis effect size (LEfSe) was contrasted based on the relative abundance of ASVs. The ASVs were picked out when alpha value of the factorial Kruskal–Wallis test was < 0.05, and the logarithmic LDA score was > 2.0. Then, the ASVs from the same genus/species were combined. The significance of the differences among the seven groups at the phylum, family, genus and ASV levels were calculated by Mann-Whitney test. The associations between the differential ASVs/bacteria and the gut barrier parameters were evaluated by Spearman’s rank correlation.

### Cecal short chain fatty acids’ measurement

The concentrations of the short chain fatty acids (SCFAs), including acetate, propionate, butyrate, isobutyrate and n-valerate, and isovalerate in the cecal content were determined as follows. Two milliliters of supernatant were prepared by reconstituting all cecal content of each mouse in 0.01 M PBS followed by centrifugation at 9000 g for 5 min at 4 °C. The supernatant was acidified with a 1/10 volume of 50% H_2_SO_4_ and extracted with ethyl ether. The concentrations of SCFAs were determined in the organic phase using an Agilent 6890 N gas chromatograph (Agilent Technologies, Wilmington, DE, USA) equipped with a polar HP-FFAP capillary column (0.25 mm × 0.25 mm × 30 m) and flame ionization detector (Agilent Technologies, Wilmington, DE, USA). Helium was used as the carrier gas. The initial oven temperature was 120 °C, which was maintained for 16 min and then raised to 122 °C at 5 °C / min, increased to 250 °C at 30 °C / min, and held at this temperature for 3 min. The detector temperature was 270 °C, and the injector temperature was 260 °C. Data handling was performed with an Agilent ChemStation (version G2070AA, Agilent Technologies, Wilmington, DE, USA).

### Statistical analysis

Normally distributed data were analyzed by analysis of variance (ANOVA) followed by LSD *post hoc* test. Data that did not meet the assumptions of analysis of variance were analyzed by the Mann-Whitney test. *P* < 0.05 was considered as statistically significant.

### Electronic supplementary material

Below is the link to the electronic supplementary material.

Supplementary Material 1

## Data Availability

The dataset for this study can be found in GenBank Sequence Read Archive database (accession number SRP323179).
