# Supplementary material for: Human-derived bacterial strains mitigate colitis via modulating gut microbiota and repairing intestinal barrier function in mice
Source: BMC Microbiol. 2024 Mar 23;24:96. doi: 10.1186/s12866-024-03216-5 (PMC10960398; doi:10.1186/s12866-024-03216-5)
Supplement: Supplementary file 1 — Supplementary Material 1 [file 12866_2024_3216_MOESM1_ESM.pdf]

Supplementary Materials

The supplementary information includes 10 supplementary figures.

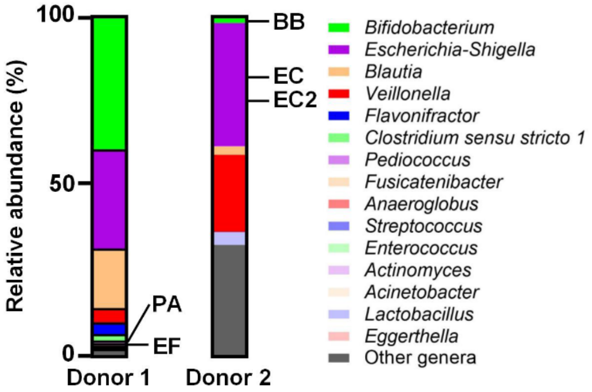

Additional file 1. Stacked bar plot of phylogenetic composition of bacterial taxa at the genus level (top 15) in fecal samples of the two donors by 16S rRNA gene sequencing. PA: *Pediococcus acidilactici*, EF: *Enterococcus faecium*, EC: *Escherichia coli*, BB: *Bifidobacterium bifidus*, EC2: *Escherichia coli* 2.

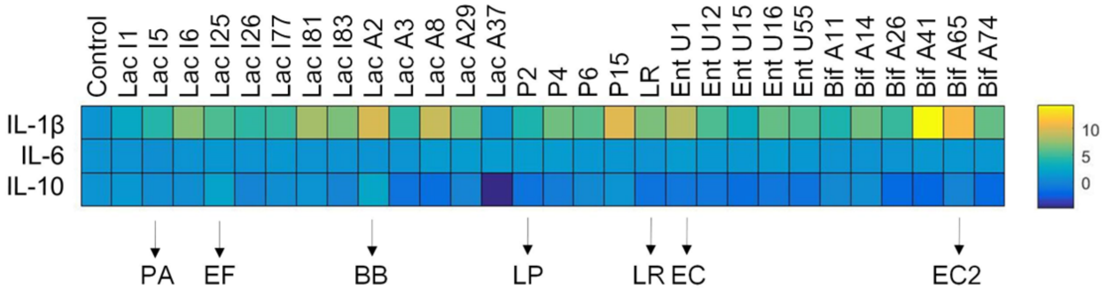

Additional file 2. Screening of the anti-inflammatory effects of the 29 bacterial strains according to ELISA analysis. LR: *Lactobacillus rhamnosus*, LP: *Lactobacillus plantarum*, PA: *Pediococcus acidilactici*, EF: *Enterococcus faecium*, EC: *Escherichia coli*, BB: *Bifidobacterium bifidus*, EC2: *Escherichia coli* 2.

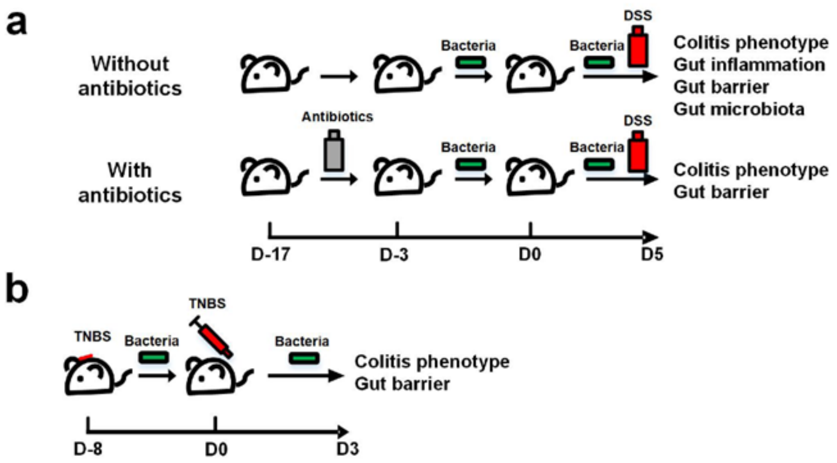

Additional file 3. Schematic diagram of colitis models. (A) Procedure of DSS-induced colitis pretreated with/without antibiotics and further treated by selected strains. (B) Procedure of TNBS-induced colitis and intervention by selected strains.

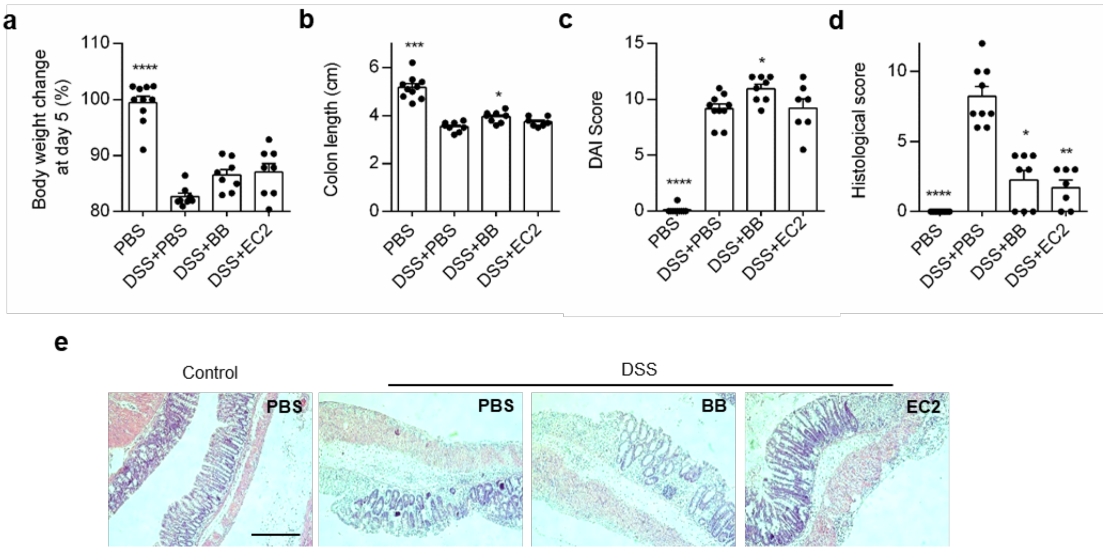

Additional file 4. 2 human-derived bacteria strains failed to reduce the overall severity of DSS-induced colitis *in vivo*. (A) Percentage of body weight changes at day 5 after DSS administration. (B) Colon length of mice at day 5 after DSS administration. (C) Quantification of the DAI score of mice at day 5 after DSS administration. (D) Quantification of histological scores of colon tissue. E) Representative images showing H&E staining of DSS-induced colitis tissue in mice. Scale bar = 100μm. *n* = 5-10 mice pr group. *P < 0.05, **P < 0.01, ***P < 0.001, ****P < 0.0001 vs DSS+PBS group by ANOVA followed by LSD post hoc test. BB: *Bifidobacterium bifidus*, EC2: *Escherichia*

coli 2.

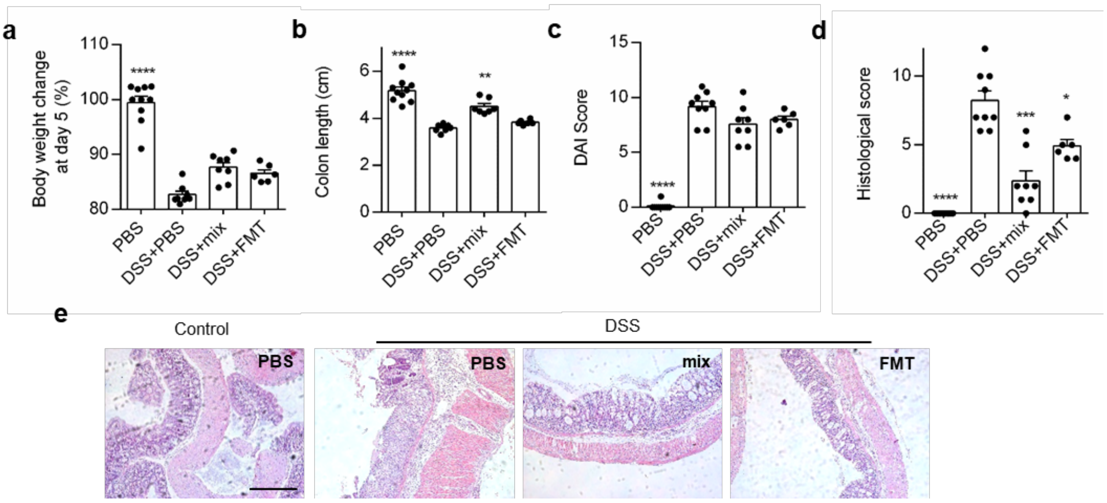

Additional file 5. Effect of mixed gut bacteria and FMT administration on DSS-induced colitis mice. (A) Percentage of body weight changes at day 5 after DSS administration. (B) Colon length of mice at day 5 after DSS administration. (C) Quantification of the DAI score of mice at day 5 after DSS administration. (D) Quantification of histological scores of colon tissue. (E) Representative images showing H&E staining of colonic tissue in DSS-induced colitis mice. Scale bar = 100μm. *n* = 5-10 mice pr group. *P < 0.05, **P < 0.01, ***P < 0.001, ****P < 0.0001 vs DSS+PBS group by ANOVA followed by LSD post hoc test.

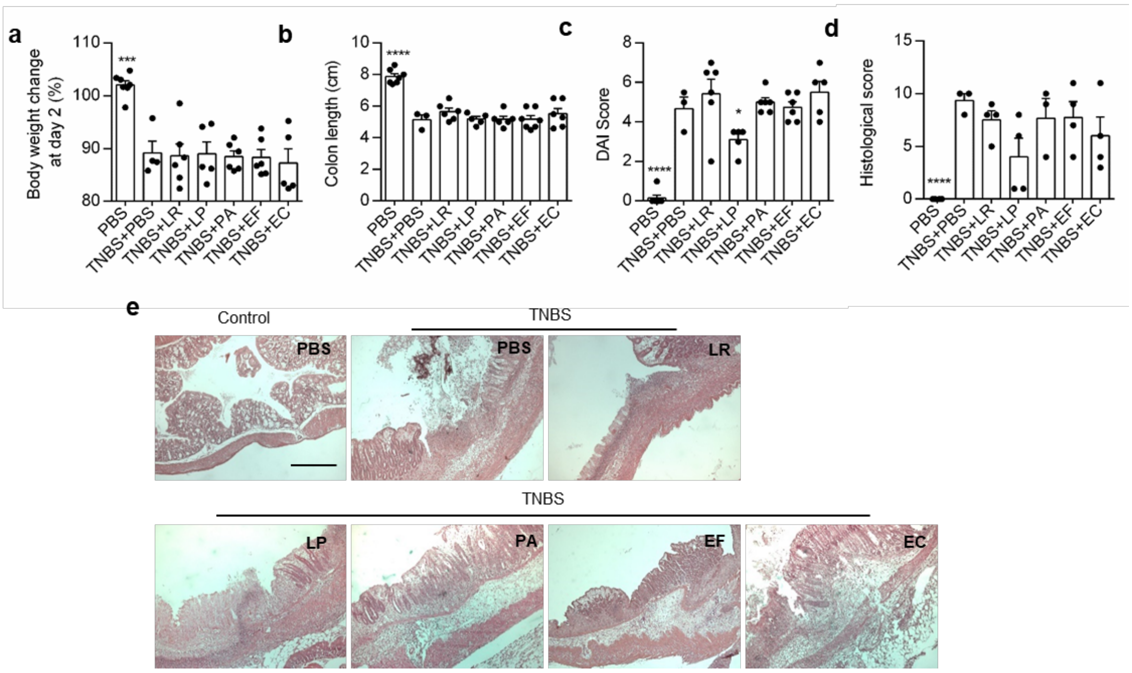

Additional file 6. Human-derived bacteria strains failed to alleviate the severity of TNBS-induced colitis *in vivo*. (A) Percentage of body weight changes at day 2 after TNBS administration. (B) Colon length of mice at day 2 after TNBS administration. (C) Quantification of the DAI score of mice at day 2 after TNBS administration. (D) Quantification of histological scores of colon tissue. (E) Representative images showing H&E staining of TNBS-induced colitis tissue in mice. Scale bar = 100µm. *n* = 5-10 mice pr group. *P < 0.05, ***P < 0.001, ****P < 0.0001 vs DSS+PBS group by ANOVA followed by LSD post hoc test. LR: *Lactobacillus rhamnosus*, LP: *Lactobacillus plantarum*, PA: *Pediococcus acidilactici*, EF: *Enterococcus faecium*, EC: *Escherichia coli*.

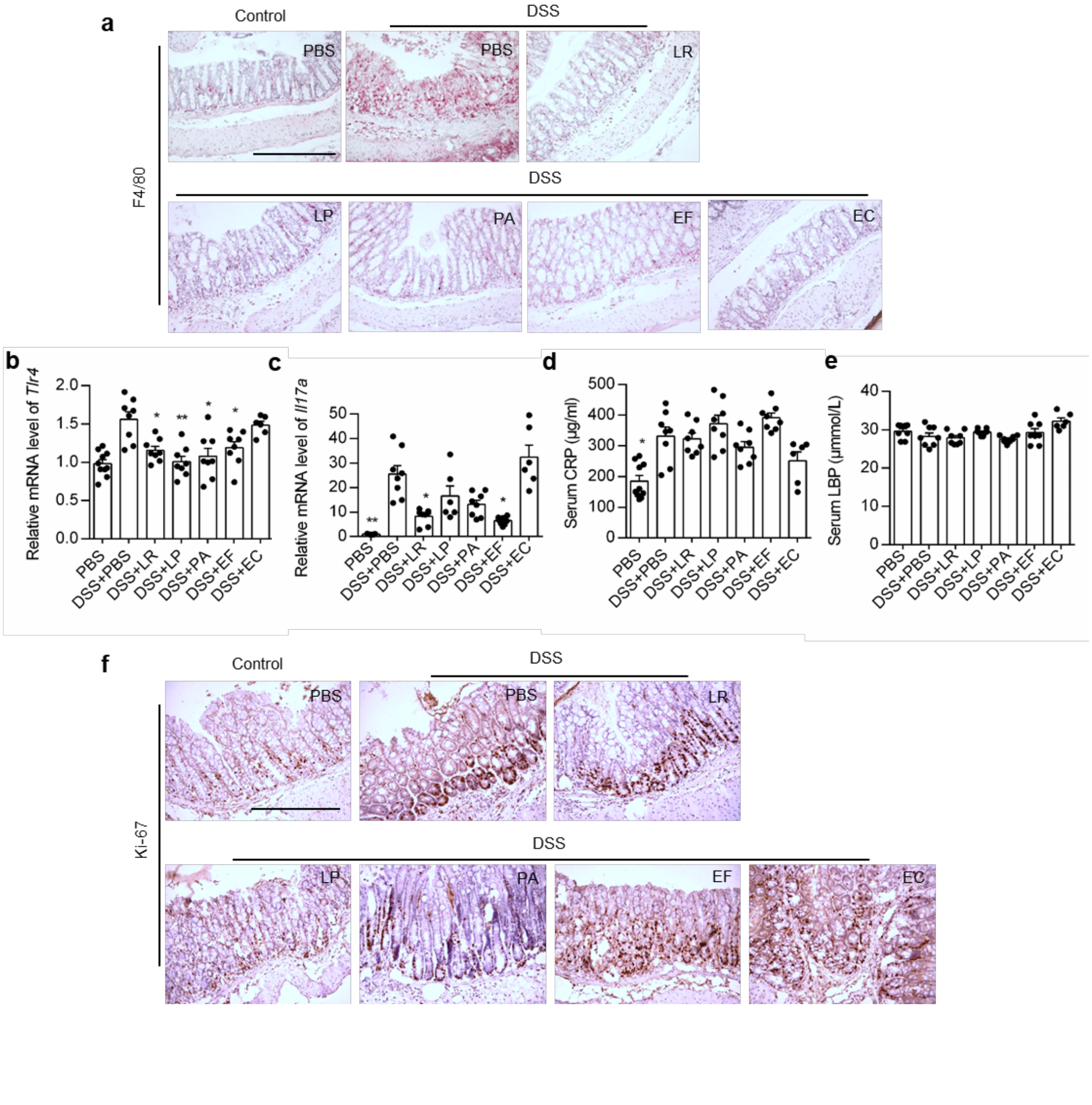

Additional file 7. Effect of human-derived bacteria on inflammatory response and proliferation of colon tissue. (A) Representative images showing immunohistological staining of F4/80 in colonic tissue of colitis mice. Scale bar = 100μm. (B) qPCR quantification of *Tlr4* expression in DSS-induced colitis tissue. (C) qPCR quantification of *Il17a* expression in DSS-induced colitis tissue. *Gapdh* served as an endogenous control. (D and E) Serum CRP (D) and LBP (E) level determined by ELISA. (F) Representative images showing Ki-67 staining of colonic tissue in DSS-induced colitis mice. Scale bar = 100μm. n = 5-10 mice pr group. *P < 0.05, **P < 0.01 vs DSS+PBS group by ANOVA followed by LSD post hoc test. LR: *Lactobacillus rhamnosus*, LP: *Lactobacillus plantarum*, PA: *Pediococcus acidilactici*, EF: *Enterococcus faecium*, EC: *Escherichia coli*.

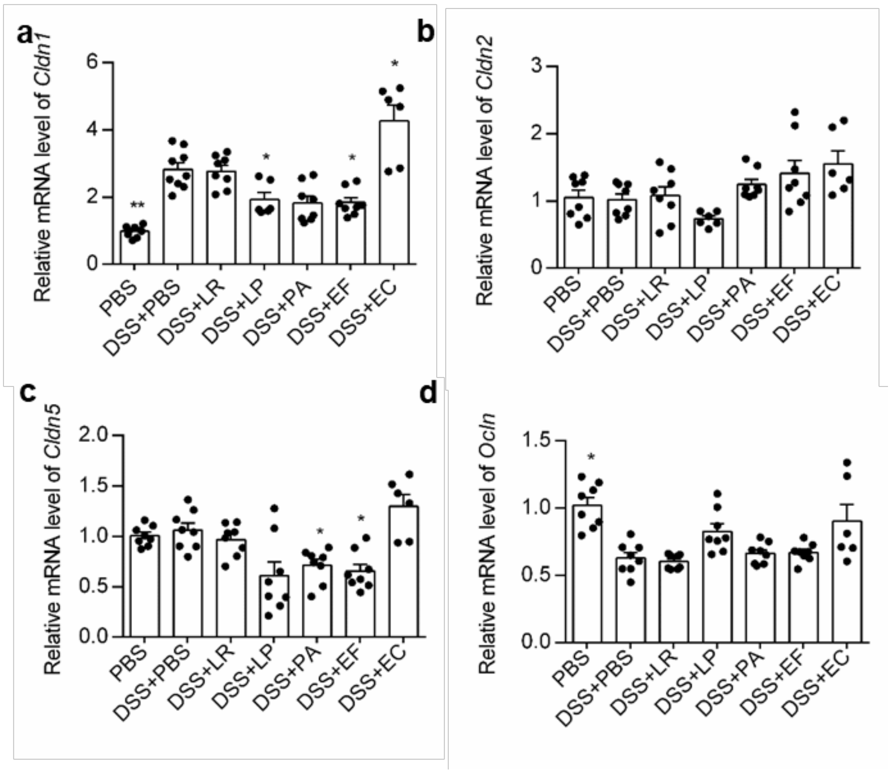

Figure S8. Expression of tight junction proteins. (A-D) qPCR quantification of *Cldn1* (A), *Cldn2* (B), *Cldn5* (C) and *Ocln* (D) expression colonic tissue in DSS-induced colitis mice. *n* = 5-10 mice pr group. *P < 0.05, **P < 0.01 vs DSS+PBS group by ANOVA followed by LSD post hoc test. LR: *Lactobacillus rhamnosus*, LP: *Lactobacillus plantarum*, PA: *Pediococcus acidilactici*, EF: *Enterococcus faecium*, EC: *Escherichia coli*.

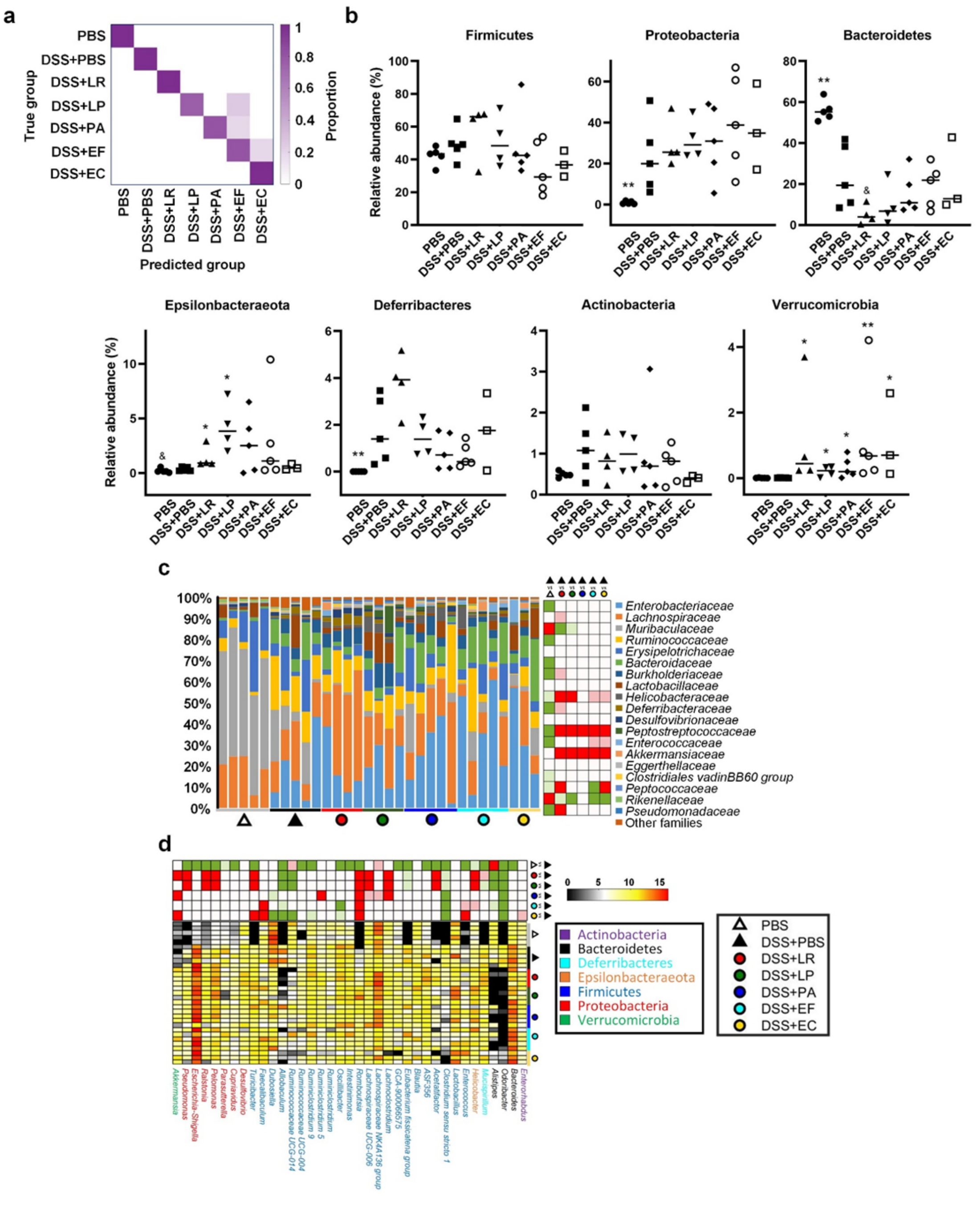

Figure S9. Human gut bacteria strains modulated gut microbiota structure in the phylum, family and genus levels. (A) Correlation between predicted group by random forest and true group. (B) The relative abundance of the seven phyla (>0.1% abundance). In the plot, the dot means every mice. The line marked the median. (C) Stacked bar plot of phylogenetic composition of bacterial taxa at the family level (19 families, >0.1% abundance) (left). The statistical significance of difference of these families between

the other group and the DSS+PBS group by Mann-Whitney (right). Dark/light green represented the taxa was decreased with p<0.05/0.1, while dark/light red represented the taxa was increased with p<0.05/0.1 compared with the DSS+PBS group. (D) Heatmap of 37 genera abundances (bottom). The statistical significance of difference of these genera between the other group and the DSS+PBS group by Mann-Whitney (upper). LR: *Lactobacillus rhamnosus*, LP: *Lactobacillus plantarum*, PA: *Pediococcus acidilactici*, EF: *Enterococcus faecium*, EC: *Escherichia coli*.

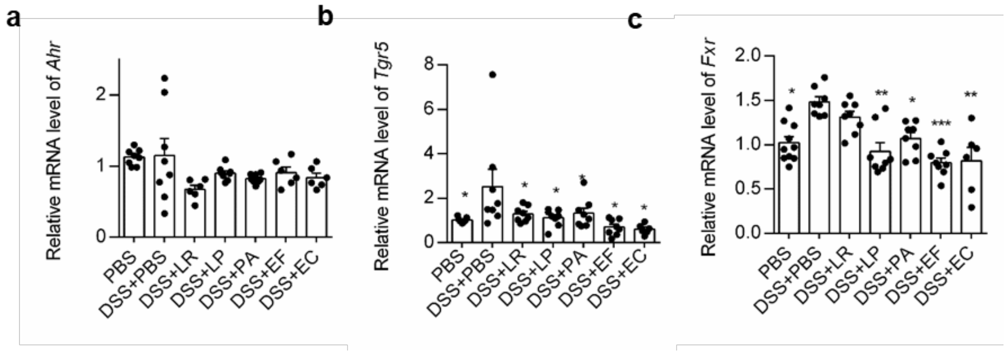

Figure S10. Expression of metabolites related receptors. qPCR quantification of *Ahr* (A), *Tgr5* (B) and *Fxr* (C) expression in colonic tissue of DSS-induced colitis mice. *n* = 5-10 mice pr group. *P < 0.05, **P < 0.01, ***P < 0.001 vs DSS+PBS group by ANOVA followed by LSD post hoc test. LR: *Lactobacillus rhamnosus*, LP: *Lactobacillus plantarum*, PA: *Pediococcus acidilactici*, EF: *Enterococcus faecium*, EC: *Escherichia coli*.
